# Isochrony in barks of Cape fur seal (*Arctocephalus pusillus pusillus*) pups and adults

**DOI:** 10.1002/ece3.11085

**Published:** 2024-03-07

**Authors:** Anna N. Osiecka, Jack Fearey, Andrea Ravignani, Lara S. Burchardt

**Affiliations:** ^1^ Department of Vertebrate Ecology and Zoology, Faculty of Biology University of Gdańsk Gdańsk Poland; ^2^ Behavioural Ecology Group, Section for Ecology and Evolution, Department of Biology University of Copenhagen Copenhagen Denmark; ^3^ Sea Search Research and Conservation NPC Cape Town South Africa; ^4^ Department of Statistical Sciences, Centre for Statistics in Ecology, Environment and Conservation University of Cape Town Cape Town Western Cape South Africa; ^5^ Comparative Bioacoustics Group Max Planck Institute for Psycholinguistics Nijmegen The Netherlands; ^6^ Center for Music in the Brain, Department of Clinical Medicine Aarhus University Aarhus C Denmark; ^7^ Department of Human Neurosciences Sapienza University of Rome Rome Italy; ^8^ Leibniz‐Zentrum Allgemeine Sprachwissenschaft Berlin Germany

**Keywords:** beat precision, pinniped, rhythm production, vocal communication, vocal repertoire

## Abstract

Animal vocal communication often relies on call sequences. The temporal patterns of such sequences can be adjusted to other callers, follow complex rhythmic structures or exhibit a metronome‐like pattern (i.e., isochronous). How regular are the temporal patterns in animal signals, and what influences their precision? If present, are rhythms already there early in ontogeny? Here, we describe an exploratory study of Cape fur seal (*Arctocephalus pusillus pusillus*) barks—a vocalisation type produced across many pinniped species in rhythmic, percussive bouts. This study is the first quantitative description of barking in Cape fur seal pups. We analysed the rhythmic structures of spontaneous barking bouts of pups and adult females from the breeding colony in Cape Cross, Namibia. Barks of adult females exhibited isochrony, that is they were produced at fairly regular points in time. Instead, intervals between pup barks were more variable, that is skipping a bark in the isochronous series occasionally. In both age classes, beat precision, that is how well the barks followed a perfect template, was worse when barking at higher rates. Differences could be explained by physiological factors, such as respiration or arousal. Whether, and how, isochrony develops in this species remains an open question. This study provides evidence towards a rhythmic production of barks in Cape fur seal pups and lays the groundwork for future studies to investigate the development of rhythm using multidimensional metrics.

## Graphical abstract

We provide the first recordings of pups barking in cape fur seals. Furthermore, we show that cape fur seal barks in both pups and adults show metronome‐like patterns. While adults produce very consistent sequences, matching theoretical beats well and showing isochrony, pups leave more breaks between barks but are already good at matching a theoretical beat sequence. Both age classes showed a worse beat precision when barking at higher rates.

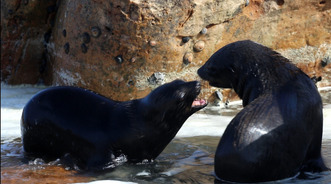

## 1 INTRODUCTION

How do acoustic communication signals evolve in humans and other animals? To address this question, a cross‐species ontogenetic angle is key: One needs to track various parameters characterising acoustic communication throughout development within a species (Fehér et al., 2017; Kocsis et al., 2024; Langehennig‐Peristenidou et al., 2023; Raimondi et al., 2021; Taylor et al., 2021). Are these parameters tuned differently across age classes, and how rhythmic can animals be at an early age? Only once we have a comprehensive overview of rhythmic parameters, across species and age classes, will we be able to uncover an often‐overlooked dimension of acoustic communication: rhythm and timing (Criscuolo et al., 2023; Kotz et al., 2018).

Traditionally, a focus of bioacoustics has been on spectral parameters, such as fundamental frequency (Bradbury & Vehrencamp, 2011; Briefer, 2012; Favaro et al., 2014; Knörnschild et al., 2012; Martin et al., 2021a). In contrast, the temporal structure of acoustic signals is a dimension of bioacoustics receiving increasing interest (e.g., Greenfield et al., 2021; Ravignani, 2019a). Regular, rhythmic patterns are fairly common, but what influences the temporal structure of an acoustic signal? Factors that have already been identified include, among others, genetics (Hoy, 1974) body size (Jichao et al., 2012) and cultural learning (Noad et al., 2000). These factors can inform, for example on the callers' species (Hoy, 1974), individual identity (Mathevon et al., 2017), quality (van den Broek & Todd, 2009) or arousal status (Martin, Gridley, Elwen, et al., 2022; Schusterman, 1977).

Three main approaches can be used to characterise acoustic rhythms in animal studies: (a) observing the spontaneous rhythm production in individuals, (b) observing the spontaneous rhythm production in groups or (c) performing playback and synchronisation experiments (Kotz et al., 2018; Ravignani, 2019a). Within the first approach, most studies focus on a specific age class when analysing rhythm production (Norton & Scharff, 2016; Ravignani, Kello, et al., 2019), while not much is known about the development of rhythms through the course of vocal ontogeny. It has been shown that temporal structure is important in the babbling of the bat *Saccopteryx bilineata* (Fernandez et al., 2021), and that we do see changes in rhythm production, for example in indris (*Indri indri*; de Gregorio, Carugati, et al., 2021) and harbour seal (*Phoca vitulina*) pups, within their first few weeks of life (Anichini et al., 2023; Ravignani, 2019b; Ravignani, Kello, et al., 2019). Additionally, there is evidence of changes in the temporal structure of zebra finch song during vocal ontogeny (Saar & Mitra, 2008). Focusing on the observational approach, one could compare rhythm production in the same species across age classes, to understand whether rhythmic patterns are already present in a given species at an early age. This is what we attempt here in pinnipeds.

Pinniped vocalisations, depending on the species, are moderately well‐studied. They are sometimes characterised as rhythmic, making them an interesting model for investigating vocal rhythms (Mathevon et al., 2017; Ravignani et al., 2016). For example, eared seals use rhythmic barking in a variety of contexts, for example as a mild threat to prevent direct physical conflict (Attard et al., 2010; Martin, Gridley, Elwen, et al., 2022; Schusterman, 1977). Adult Otariid barking consists of a series of short percussive vocalisations in a highly metronome‐like, that is isochronous, pattern (Martin et al., 2021b; Martin, Gridley, Elwen et al., 2022; Phillips & Stirling, 2001; Schusterman, 1977). The barks' spectral structure can inform about the caller's identity (Attard et al., 2010), and barks' production rates can convey arousal levels (Martin, Gridley, Elwen, et al., 2022; Schusterman, 1977). Nevertheless, descriptions of vocal repertoires of Otariids tend to focus on the young to mother‐and‐pup communication, which does not include barking (e.g., Charrier, 2021; Martin et al., 2021b; Phillips & Stirling, 2001; Stirling, 1972; Tripovich et al., 2008), or adults' reaction to pups (Phillips & Stirling, 2001). While barks have previously been observed in Cape fur seal (*Arctocephalus pusillus pusillus*) pups (Martin et al., 2021b), they were only recorded and described in adults (Martin et al., 2021b; Martin, Gridley, Elwen, et al., 2022). This is likely because pup vocalisations outside of the interactions with their mothers are more difficult to encounter and record. As a result, even though the behaviour of a pinniped pup is not limited to interaction with its mother (Farentinos, 2010; Gentry, 1974), nearly nothing is known about barking in pups or their vocal development (Khan et al., 2006). In particular, we ask, are temporal patterns of vocalisations already present at an early age?

Here, we present an exploratory study probing for the presence of isochronous barking in the Otariid Cape fur seal. We observed spontaneous bark production in individuals of two age classes, pups and adults. This study is the first quantitative description of barking in Cape fur seal pups. We compare the rhythm of bark production within and between adult females and unsexed pups, in contexts outside of mother–pup interactions. We test for the presence of isochrony, a purely acoustic property, versus beat, a more cognitive feature, in two age classes. Note that here and throughout the text, when using the terms ‘isochrony’, ‘beat’ or ‘beat‐keeping’, we refer to vocal production, rather than sound perception (Cook et al., 2013; Rouse et al., 2016).

## 2 METHODS

### 2.1 Site and set‐up

Between 3 April and 9 April 2019, we collected acoustic and behavioural data on the Cape fur seal (*Arctocephalus pusillus pusillus*) mothers and pups at Cape Cross Seal Reserve, Namibia (21°48′ S, 14°1′ E), during an exploratory project on mother–pup communication (Osiecka et al., 2020, 2022). At that time of year, most pups are approximately 4 months old and still completely dependent on their mothers for food (Kirkman & Arnould, 2018) yet have begun to undertake short exploration trips into the colony and shallow waters. Focal‐follow recordings were taken with a TASCAM DR‐680M portable multitrack recorder (20 Hz–20 kHz, +0.5/−0.5 dB) and a VP89L shotgun condenser microphone (flat frequency response: 40 Hz–20 kHz, −33.5 dBV/Pa at 1 kHz). The microphone was attached to a ~1.5 m telescoping boom and temporally paired with video footage (GoPro 4). The observer recorded vocal notes to identify focal individuals and behavioural contexts.

We observed pups producing sequences of barks similar to those of adults (see audio samples in Appendix S1). These barks occurred either in a mock fight between a group of pups (starting between pups, or in one case after two nearby adult females barked at each other), or when a female was accompanied by a pup, joining in her vocalisations (in one instance towards another adult female). At this time of the year, only a few adult males are present at the Cape Cross colony. For this exploratory study, we opportunistically recorded pups of unknown sex during mock fights, and adult females barking at different locations throughout the colony, using vocal notes to identify bouts produced by different individuals.

### 2.2 Analysis

We selected the available recordings for an exploratory analysis, which dictated the number of barking bouts available for the pups (i.e., we managed to obtain recordings from 17 individual pups, one sequence per pup) and randomly selected an equal number of adult females barking bouts (*n* = 17, one sequence per individual). We only used pup bouts produced during mock fights and female bouts produced during apparent mild threat displays. We then extracted the temporal information from these calls, that is the time of the beginning and end of each vocalisation in a bout (Ravignani, 2018a), using Raven Pro 1.6.4 (Cornell Lab of Ornithology, 2022), which was also used to calculate inter‐onset intervals (IOI), defined as the time between the start of one vocalisation and the start of the next vocalisation (Burchardt & Knörnschild, 2020). By plotting each barking bout as an event series starting from the first vocalisation, we visually assessed the rhythmic structure of these calls (Eaton et al., 2022; Ravignani & Norton, 2017).

Different methods exist to determine the rhythm of a vocalisation sequence. We chose to transform the average IOI duration of a sequence into a frequency, or rate, by calculating $\text{Frequency}=\frac{1}{\text{Average}\;\left(\mathrm{IOI}\right)}$. This value indicates how many elements were expected per second and could be called the beat frequency best describing the sequence (Burchardt & Knörnschild, 2020). Here and throughout, we understand “beat” as the descriptor of the rhythm and as a single element in this theoretical rhythm. That way “missing a beat” indicates when a single element (in this case a bark) predicted by an isochronous pattern fails to occur in the sequence (and the predicted, but not present element is referred to as a “silent beat”). Beat precision (bp) is defined as the deviation of a single element, in this case a bark, from the predicted element timing, based on the calculated beat frequency (rhythm).

Beat‐precision values are generally calculated per element in a sequence, matching each element to the closest theoretical beat in an expected time series. The expected time series in our case was calculated using the IOI beats, we summarised the bp values per sequence. To indicate the variability of beat precision within a sequence, we also report the coefficient of variation (CV) and the normalised pairwise variability indices (nPVI) of individual beat precision values of a sequence, corresponding to their use on the IOIs of a sequence.

To describe the temporal structure of the bark sequences in adult females and pups of unknown sex, we calculated the following indices: (1) IOI duration; (2) integer ratios (de Gregorio et al., 2023; de Gregorio, Valente, et al., 2021; Demartsev et al., 2022; Jacoby & McDermott, 2017; Lameira et al., 2024; Ma et al., 2024; i.e., IOI ratios calculated for all pairs of consecutive calls in a bark sequence); (3) coefficient of variation (CV) of IOIs, adjusted for small sample sizes (Kello et al., 2017); (4) normalised Pairwise Variability Indices (nPVI; Burchardt & Knörnschild, 2020; Daniele & Patel, 2013; Grabe & Low, 2002) based on IOIs, which indicates the rhythmic structure of a sequence; (5) IOI beat in hertz (Burchardt & Knörnschild, 2020); (6) average beat precision per sequence (Burchardt et al., 2021), calculated for each element in a sequence, which indicates how well elements follow an expected time series, that is the expected pattern calculated in (2); (7) CV of beat precision values per sequence; and (8) nPVI of beat precision values per sequence. IOI durations and integer ratio values were of sufficient sample size (i.e., >30) to use a parametric test, namely Welch's unpaired *t*‐test to compare the two age classes. For all other parameters, a non‐parametric (Mann–Whitney‐*U*) test was used. Each test used a different dependent variable, and no group was tested twice; hence, *p*‐value adjustments for multiple testing were not necessary. Additionally, we ran a linear model testing the influence of the IOI beat in Hz on beat precision, pooling barking bouts of pups and adults together, and a linear mixed model testing the influence of individual mean bark duration on beat precision, controlling for the age class. All analyses were run in R 4.2.2 (R Core Team, 2022).

## 3 RESULTS

**FIGURE 1:**
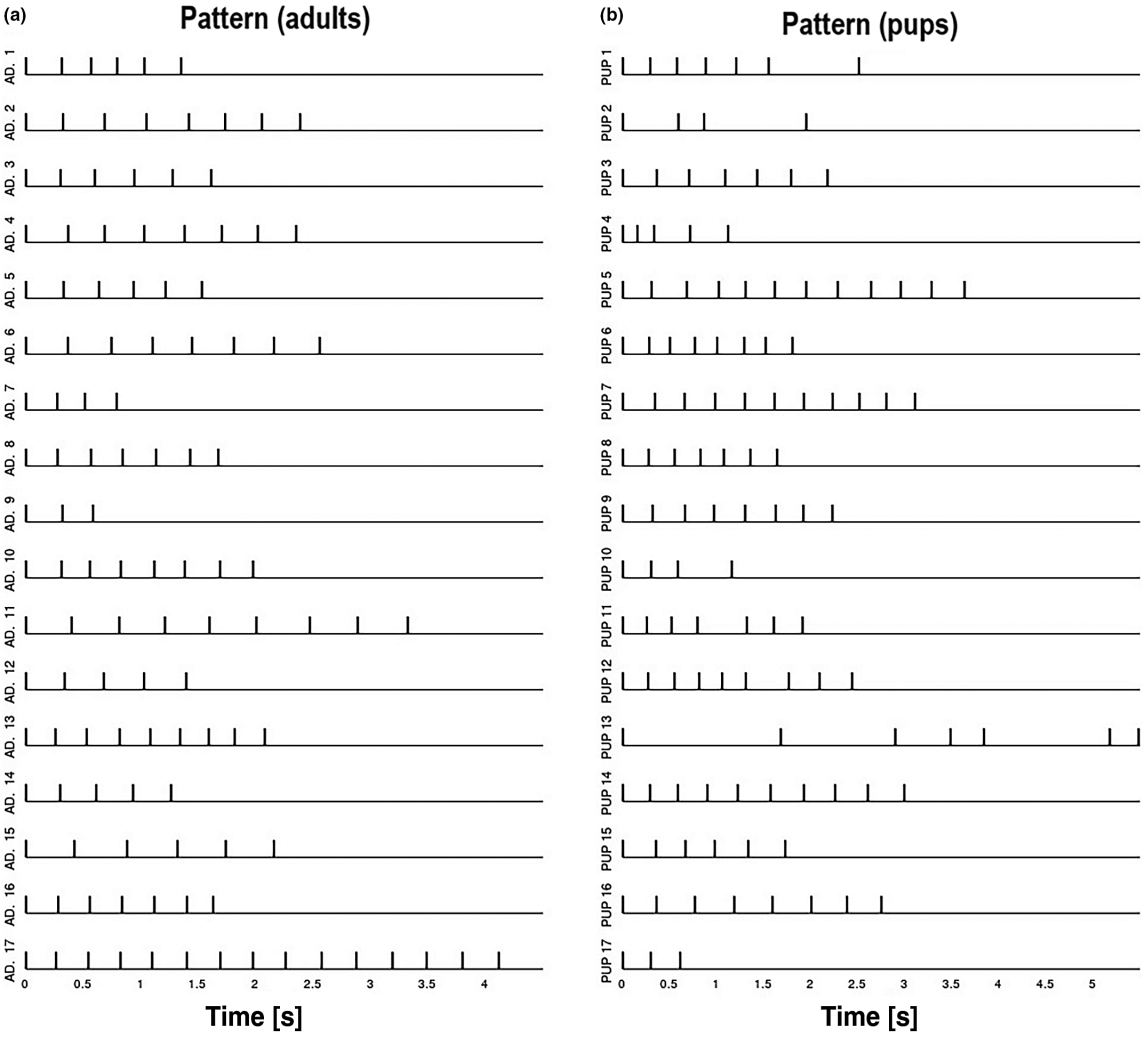
Individual patterns of bark onsets in (a) 17 adult females and (b) 17 pups. While some animals show isochronous call sequences, patterning seems to show less consistency in pups than in adults.

**FIGURE 2:**
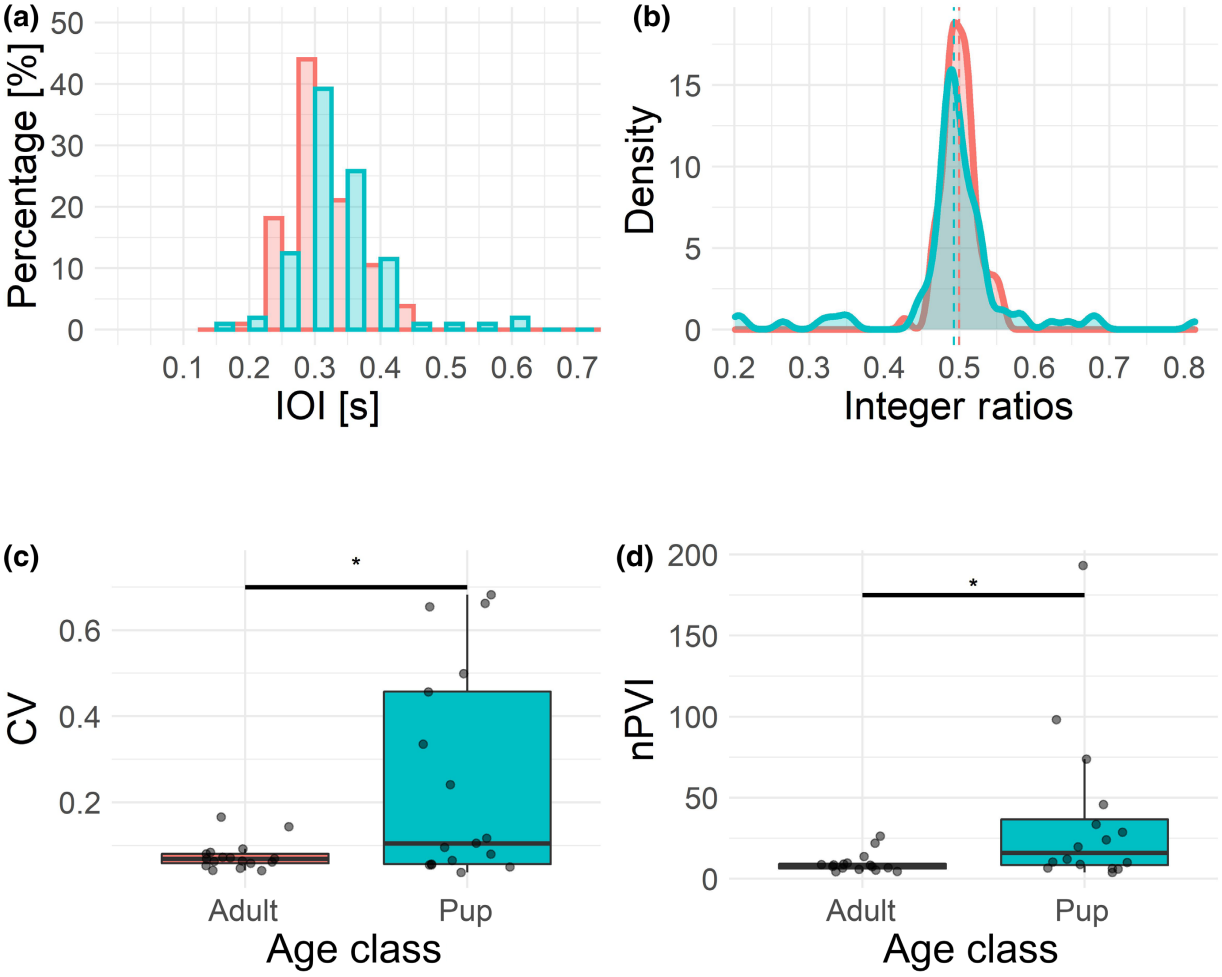
Boxplots and actual values of the four isochrony‐related variables in adults (red) and pups (blue). (a) Histogram of IOI durations showing similar distributions with slightly higher variability in pup IOIs. (b) Density plot of rhythm ratios showing a higher variability in pups (the blue distribution is less ‘peaked’ than the red one). More variability in pups is also visible in (c) the CV of IOI durations and (d) the nPVI: Both these variables are significantly higher in pups than adults. Significance level: *p < .05.

Visual inspection of the IOIs suggests that all barks produced by adult females followed an isochronous pattern (Figures 1a and 2a). While this was true for some of the barking bouts of the pups, these displayed a more jittered distribution and less consistent interval duration (Figures 1b and 2a). While nPVI values for adults indicate isochrony (Figure 2d, Table S1), these values are significantly higher for pups (Table 1, Figure 2d, Table S1). Pups also showed a significantly higher coefficient of variation of interval durations (Table 1, Figure 2c, Table S1).

**TABLE 1:** Mean values of calculated rhythm indices for adult and pup barks and statistical comparison of both age classes.

Parameter	Adults	Pups	Statistics	Meaning
IOI duration (s)	0.32	0.37	*t* = 2.4, df = 118, *p* < .05*	On average longer intervals in pups
Integer ratios	0.45	0.45	*t* = 0.77, df = 102, *p* = .44 (not significant)	Similar for pups and adults
Unbiased CV	0.08	0.25	*W* = 64, *p* = .015*	IOIs more variable in pups
nPVI	9.68	36.33	*W* = 68, *p* = .023*	Adults more isochronous
IOI beat (Hz)	3.22	2.88	*W* = 162, *p* = .21 (not significant)	Statistically similar for pups and adults
Beat precision	0.61	0.5	*W* = 170, *p* = .12 (not significant)	Statistically similar for pups and adults, with slightly less deviation from the predicted pattern in pups
CV of beat precision	0.5	0.72	*W* = 82, *p* = .08 (not significant)	Statistically similar, with slightly more variability in pups
nPVI of beat precision	38.15	90.02	*W* = 75, *p* = .046*	More variable in pups

*Note*: Significance level: *p < .05.

The integer ratios did not significantly differ between pup and adult barking sequences (Table 1, Table S2). Visually, distributions of integer ratios hint at a subtle difference: while adult barks follow an isochronous pattern, pups show some deviations from it (Figure 2b).

**FIGURE 3:**
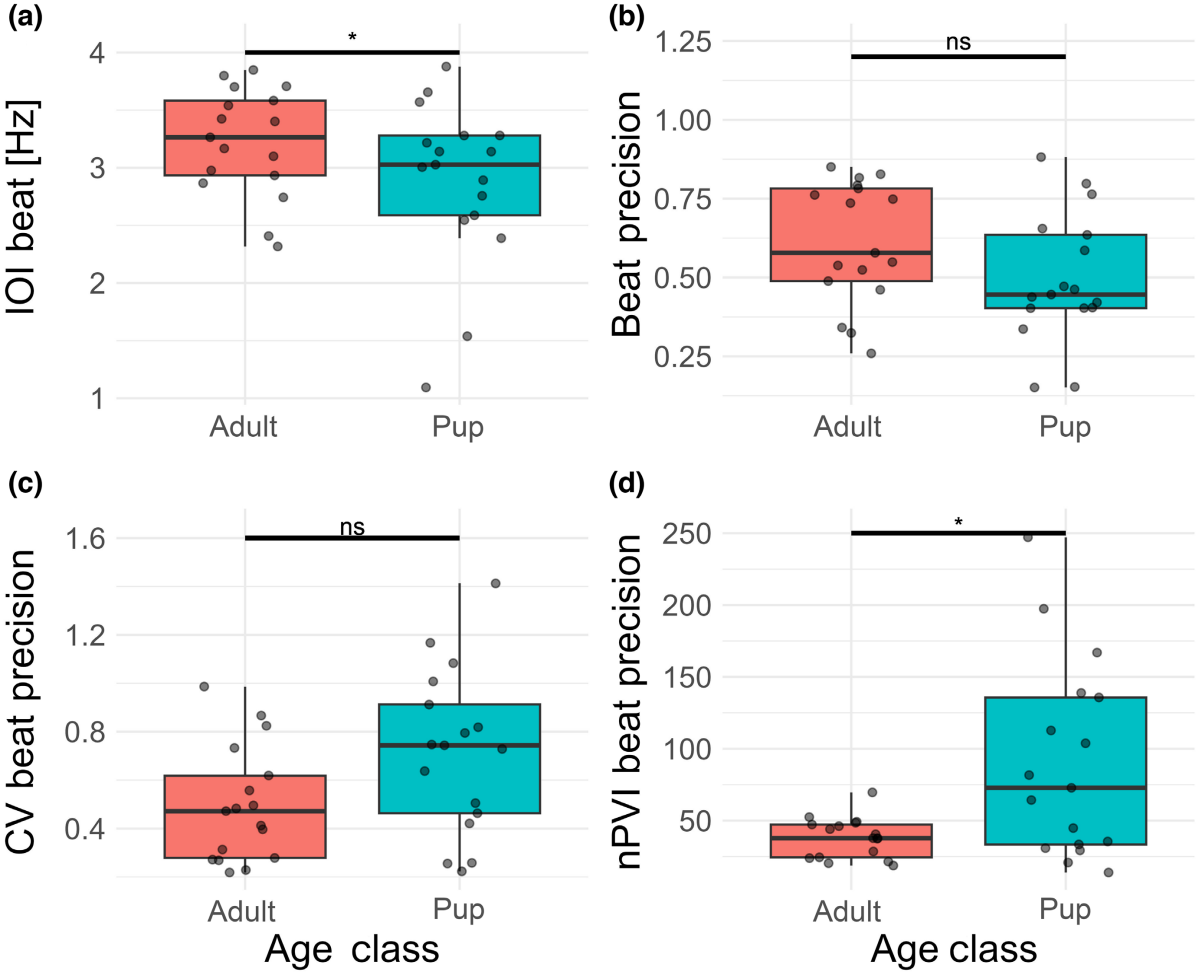
Boxplots and actual values of the four beat‐related variables in adults (red) and pups (blue). (a) Adults and pups produce sequences with fairly similar underlying beats. The age difference seems to lie in how well the beat is kept: (b) in fact, on average the beat precision values are lower in pups than in adults, that is they show a smaller deviation from the perfect template, although this relationship is not statistically significant. (c) CV of beat precision although apparently higher in pups, does not statistically differ between age classes. (d) nPVI of beat precision is significantly higher in pups, indicating a stronger deviation from a perfect isochronous template in puppyhood. Significance level: *p < .05.

Beat frequencies were similar between adults and pups (Table 1, Figure 3a, Table S1). Similarly, there was no significant difference in IOI beat precision between age classes (showing a possible trend for better beat precision in pups; Table 1, Figure 3b, Table S1).

The coefficient of variation of beat precision was slightly higher for pups than adults (Table 1, Figure 3c, Table S1). Also, the nPVIs of beat precision were significantly higher for pups (Table 1, Figure 3d, Table S1).

**FIGURE 4:**
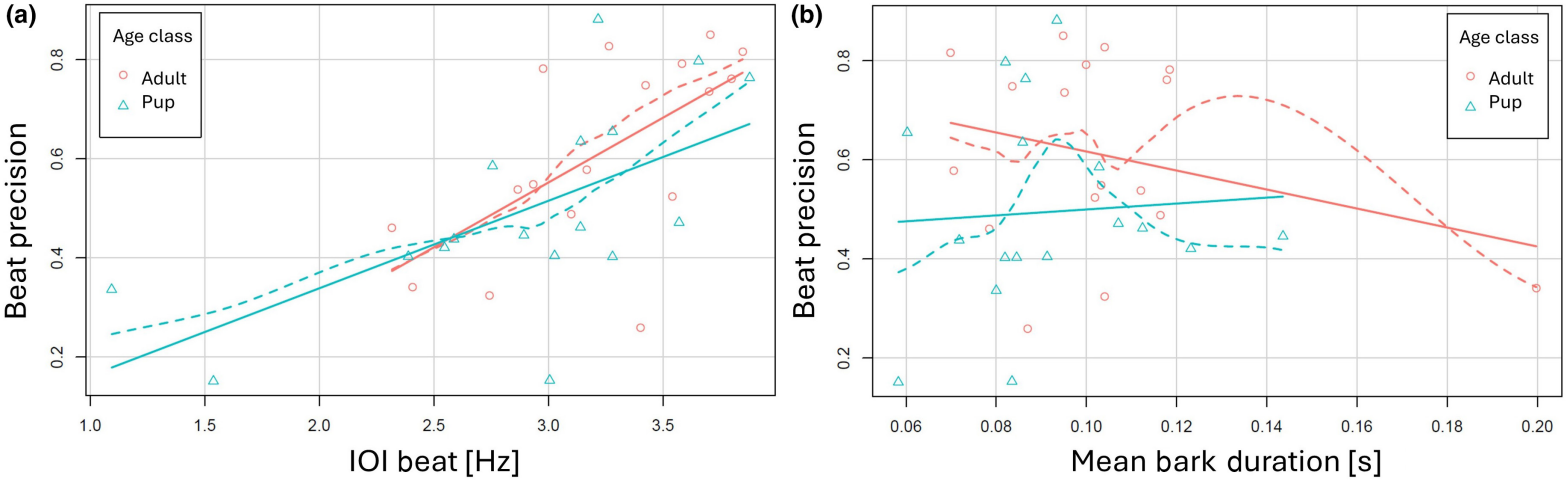
Regression plots of the linear mixed models, testing whether beat precision can be statistically explained by other rhythmic or temporal variables. (a) There is a clear effect of IOI beat on beat precision: Higher, that is faster IOI Beats lead to worse beat precision, and the effect is very similar for both age classes. (b) There is no detectable effect of mean bark duration on Beat precision. The solid lines represent the overall fitted regression line, and the broken lines are the fitted regression line for the random effect (age class). Mean bark duration does not statistically explain beat precision and shows similar values between age classes.

The IOI beat (Hz) had a significant influence on the beat precision: the faster the beat was, the lower its precision, that is the higher the value of this index (Table 2, Figure 4a).

**TABLE 2:** Results of the linear model investigating the influence of IOI beat (Hz) on beat precision.

	Predictors		Scaled residuals					*p*‐Value	Interpretation
	Intercept	IOI beat (Hz)	Min	1Q	Median	3Q	Max		
Beat precision									
Estimates	−0.098	0.213	−0.390	−0.078	0.023	0.096	0.294	>.001	The faster the beat, the lower its precision
Std. error	0.139	0.045							
*t*‐Value	−0.700	4.761							

The mean bark duration of an individual did not influence their beat precision (Table 3, Figure 4b).

**TABLE 3:** Results of the linear mixed model investigating the influence of an individual's mean bark duration on their beat precision.

	Predictors		Scaled residuals					*p*‐Value	Interpretation
	Intercept	Bark duration (s)	Min	1Q	Median	3Q	Max		
Beat precision									
Estimates	0.635	−0.845	−1.945	−0.568	−0.186	0.856	1.852	.17	No impact of the bark duration on beat precision
Std. error	0.147	1.357							
*t*‐Value	4.507	−0.618							

## 4 DISCUSSION

Here, we provided the first quantitative description of rhythmic patterns in barking bouts of the Cape fur seal. We also analysed the first recordings of barking in pups—a vocalisation type previously mentioned for pups but only analysed in adults of this species (both male and female; Martin et al., 2021b; Martin, Gridley, Elwen, et al., 2022). We investigated the rhythm of spontaneous bark production in 17 pups of unknown sex and 17 adult females in their natural environment. Our study shows that while both age classes bark in an isochronous rhythm, there seems to be variation between the precision of that rhythm and the success of producing a vocalisation on every beat. We also show that barking rates potentially influence beat precision in both age classes.

As this study builds on a rather small sample size, we understand there may be concerns regarding the strength and interpretability of the results of this data set. Due to the opportunistic nature of the recordings, 17 pups and 17 adult females were recorded, and only once per individual. Cape fur seal pups do not possess natural individually identifiable characteristics, and due to logistical constraints, individuals could not be dyed, limiting long‐term identification. We also did not have the chance to control for some factors of interest, such as the exact age of the barking individuals, robust indicators of arousal (e.g., heart rate), or the distance between the caller and the microphone (essential for considering the amplitude of the calls). Otariid pup barks are difficult to encounter and can be challenging to record within their colonies, which makes even this limited sample size an important and rare insight into pup vocal communication. Most of our results are descriptive, with a focus on providing the first quantitative description of Cape fur seal pup barks. Comparing them to adult female barks, even in this small sample, showed that a more detailed study of Cape fur seal pup barking would lead to valuable insights into the ontogeny of vocal rhythms, which is why we see a lot of value in this data set, even though the inference one can draw from it has limited statistical power and is of course limited. Additional data collected in a more controlled setup would be necessary for future studies to address the issues outlined in this observational study. Henceforth we focus on potential explanations and questions that could be asked in future studies to avoid over‐interpreting this data set. We hope that future collaborative research will allow combining expert fieldwork methods (Martin et al., 2021a, 2021b; Martin, Gridley, Elwen, et al., 2022; Martin, Gridley, Fourie, et al., 2022) with the multidimensional quantitative rhythmic analyses we present here and perhaps even strictly controlled experiments targeting temporal pattern perception (Hanke et al., 2021; Heinrich et al., 2020, 2022; Verga et al., 2022).

While vocalisations of adult females followed the general rule of highly rhythmic Otariid barks (Charrier, 2021; Martin, Gridley, Elwen, et al., 2022; Schusterman, 1977), pup vocalisations were slightly more erratic, as pups' bark sequences broke away from ‘signal isochrony’ more often than those of adults. The normalised pairwise variability indices and coefficients of variation displayed high values for pup barks, in both the entire sequence and on a per‐element basis in terms of beat precision. This shows that pups bark at much more variable intervals than adult females. While both groups produce barking sequences with an underlying metronome‐like beat, adult females show ‘signal isochrony’ and pup sequences show several ‘silent beats’ (Burchardt, 2021). This means that (1) adults produce a bark on every beat of a theoretical metronome‐like sequence, and (2) pups do produce barks on beats but sometimes skip several beats before producing another bark (Figure 1). Tentatively, these data may be consistent with a finite state machine model of rhythmic sequences: a rhythmic ‘state’, equivalent to the isochronous ratio, may be present already in puppyhood with transition probabilities instead changing over development (see figure 2 of Ravignani, 2018b). In summary, based on this dataset, isochrony and beat precision seemed to be different between pups and adults. We will discuss different potential reasons for that in the following paragraphs to highlight the potential of further studies.

We investigated how well individuals matched a theoretical beat in a barking sequence. We found that while pups sometimes have inconsistent ‘silent breaks’ between barks, the barks they do produce occur with high precision to a theoretical isochronous template. In other words, pups may skip some barks but when they resume, the bark falls on the precise isochronous template. Adults have a consistent barking pattern, not missing any barks in a sequence, but their barks match a theoretical isochronous template less precisely than pups (Figure 3b). Beat precision values were calculated per element, therefore we can calculate variability parameters of beat precision within one barking bout (Burchardt et al., 2021). Here, adult females show less variability than pups, meaning that while pups sometimes are perfectly on beat and sometimes very imprecise, adults tend to rarely be perfectly on beat, but also never way off. Instead, adults are always only approximate in their timing, still producing an overall stereotyped pattern. This result also highlights the methodological point of using fine‐grained rhythm metrics to deliver a nuanced picture; had we used more basic approaches to rhythmic quantification, we may have not seen any quantitative rhythmic difference between pups and adults.

**FIGURE 5:**
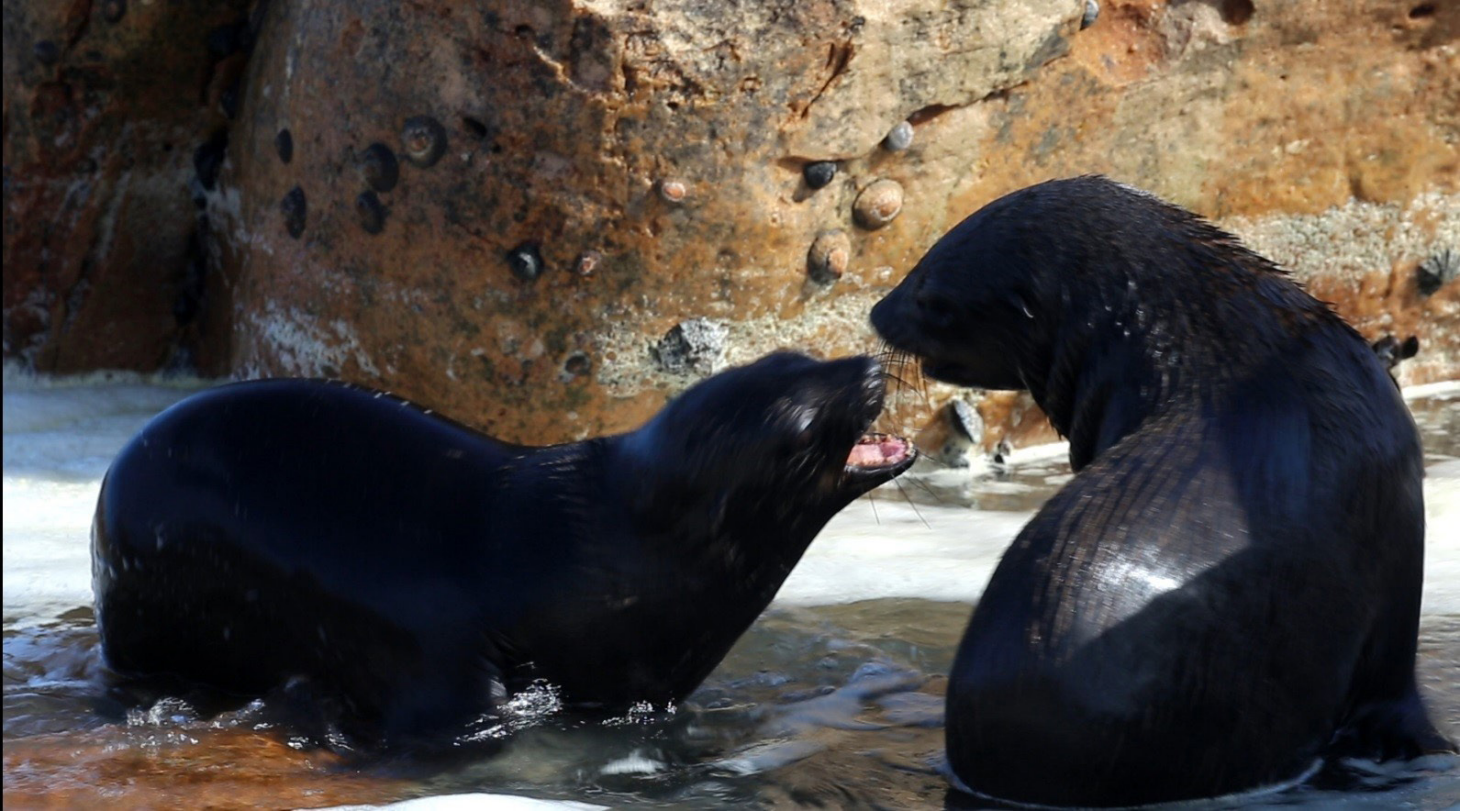
Mock fight between two pups observed at Lamberts Bay, South Africa. See Appendix S1 for a full video (courtesy of Tess Gridley).

What is the reason behind the differences between pups and adults? This cannot be answered by an observational, small sample‐size study like this one, nonetheless, we want to discuss alternative hypotheses which could drive future research. Some variance in mammalian vocalisation structure and use can be explained by factors such as growth or emotional context (Briefer, 2012; Martin, Gridley, Elwen, et al., 2022). Cape fur seal pups seem to bark mostly during mock fights with their peers (Figure 5), which prepares them for future social interaction (Farentinos, 2010; Gentry, 1974; Harcourt, 1991) that might require adequate use of barks (Martin, Gridley, Elwen, et al., 2022) and could be accompanied by higher arousal. The same is true for adults who use barks, for example in mild threat contexts (Martin, Gridley, Elwen, et al., 2022). Arousal, which is one of the basic dimensions of emotions (Russell, 1980), affects barking rates in adult Cape fur seal males (Martin, Gridley, Elwen, et al., 2022). Perhaps emotional and behavioural contexts might impact not only the rate but also the precision of vocal rhythms. Here, we observed that barking rates influenced beat precision in both pups and adult females. Since we did not have access to reliable measures of emotions (e.g., Kret et al., 2022; Mendl et al., 2010) in this observational study, we cannot conclude whether this decreased beat precision was indeed related to the animals' affective states. Future experiments in controlled conditions could answer this question.

Vocal production is highly coupled to respiration in many mammals (Pouw & Fuchs, 2022; Riede et al., 2020). While the precise matching of beats in pups could be correlated to less vocal control and therefore more control through muscle tonicity and respiration patterns, those same respiration patterns could be the reason for pups to miss beats. For example, they might be out of breath, as they are considerably smaller, which correlates to smaller lung capacities (Hermann‐Sorensen et al., 2020; Kooyman, 1989; Thometz et al., 2021). For an adult female, it might be easier to maintain a barking bout over a longer time without skipping a beat. Finally, skipping a beat can be a simple byproduct of other actions, such as biting one's mock fight companion, or dodging a bite.

We do not know whether for a Cape fur seal maintaining isochrony is inherently important (e.g., conveys socially important information such as suggested for *Nomascus* and *Hylobates* gibbons; Raimondi et al., 2023; de Gregorio et al., 2023; Ma et al., 2024), or an artefact of the most efficient way of performing this vocalisation (similar to, e.g., walking), which may require practice to develop. If maintaining a roughly isochronous pattern requires practice, a young animal may put more effort and slightly overaccentuate the rhythmic action before it becomes fluent in it.

Many animals can perceive rhythmic patterns and adjust the temporal structure of calls (Greenfield & Merker, 2023; Ravignani, Verga, et al., 2019). For example, an animal may time its vocalisations so as not to overlap with others (e.g., Allen‐Ankins & Schwarzkopf, 2021; Anichini et al., 2023; Cade & Otte, 1982; Dadour, 1989; Grafe, 1996; Ravignani, 2019b; Ravignani, Verga, et al., 2019), or reversely—synchronise their signals to mask others (Greenfield & Roizen, 1993) or signal group cohesion and regulate social interactions (Gamba et al., 2016; Raimondi et al., 2023). If we consider barks as vocal exchanges between individuals, it seems likely that the temporal structure of barking bouts might be in some way influenced by other conspecific's calls. Unfortunately, our observations of Cape fur seal barking in a noisy colony, with tens of others calling in the vicinity at any given moment, did not provide an opportunity to investigate the potential timing adjustments in barking bouts. Dedicated experiments in controlled conditions, such as playbacks in a captive setting, might help us establish whether timing adjustment takes place in this species, and contribute to our understanding of the evolution of rhythm (Greenfield & Merker, 2023; Ravignani, Dalla Bella, et al., 2019).

Our initial observations in uncontrolled conditions cannot explain why rhythmic patterns in Cape fur seal barks differ between age classes; we do, however, report multiple differences between puppyhood and adulthood. We also show that, independently of the age class, faster bark rates result in lower beat precision, which might be related to the affective state of the caller (though see figure S2 in Raimondi et al.). Further studies should aim at understanding whether our preliminary findings generalise to stable individual patterns and the whole species. Controlling for pups' age, affective state and body mass may shed light on how the rhythmic structure of barks develop in detail and contribute to our understanding of both pinniped behaviour and the development of vocal rhythms in general.

## 5 CONCLUSIONS

We provide the first recordings of pups barking in Cape fur seals. Furthermore, we show that both pups and female adult barks follow a metronome‐like rhythmic pattern. While adults produce very consistent sequences, matching theoretical beats well and showing isochrony, pups leave more breaks between barks. Still, they are already good—and potentially better than adult females—at matching a theoretical beat sequence. Both age classes showed a worse beat precision when barking at higher rates. Differences might be explained by physiological constraints, like respiration or arousal differences. This makes Cape fur seals an ideal candidate to study the development of rhythmic patterns during vocal ontogeny.

## INSTITUTIONAL REVIEW BOARD STATEMENT

The study complies with the European Union Directive on the Protection of Animals Used for Scientific Purposes (EU Directive 2010/63/EU) and current Namibian laws. Fieldwork was permitted by the Namibian Ministry of Fisheries and Marine Re‐sources to Sea Search Research and Conservation, Namibian Dolphin Project.

## AUTHOR CONTRIBUTIONS

**Anna N. Osiecka:** Conceptualization (lead); data curation (lead); investigation (equal); methodology (supporting); project administration (lead); writing – original draft (equal); writing – review and editing (lead). **Jack Fearey:** Investigation (equal); writing – review and editing (supporting). **Andrea Ravignani:** Conceptualization (supporting); funding acquisition (lead); methodology (equal); writing – review and editing (supporting). **Lara S. Burchardt:** Formal analysis (lead); methodology (equal); supervision (lead); visualization (equal); writing – original draft (equal); writing – review and editing (supporting).

## FUNDING INFORMATION

The Comparative Bioacoustics Group was funded by Max Planck Group Leader funding to A.R. The Center for Music in the Brain is funded by the Danish National Research Foundation (DNRF117). A.R. was funded by the European Union (ERC, TOHR, 101041885). LSB was funded by the DAAD.

## CONFLICT OF INTEREST STATEMENT

The authors declare no conflict of interest.

## Supporting information


Figure S1

Table S1

Table S2

Appendix S1

## Data Availability

The full data set generated in this study is available at https://osf.io/6rn82/?view_only=5687781b6424423b99b01b7c98a8af16.
