# Supplementary figures and images for: Isochrony in barks of Cape fur seal (Arctocephalus pusillus pusillus) pups and adults
Source: Ecol Evol. 2024 Mar 7;14(3):e11085. doi: 10.1002/ece3.11085 (PMC10920323; doi:10.1002/ece3.11085)

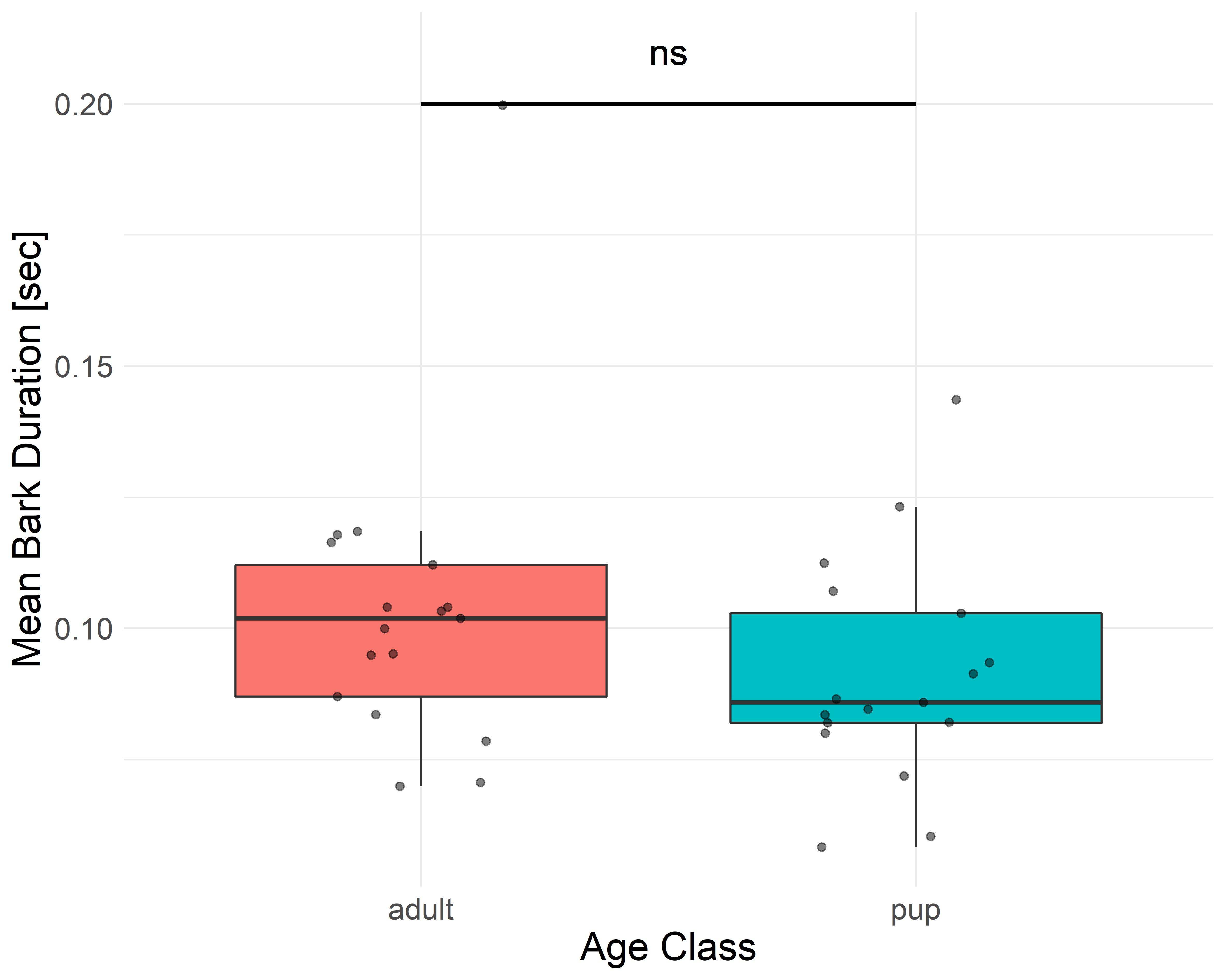

Supplement: Supplementary file 1 — Figure S1 [file ECE3-14-e11085-s003.jpg]
