# Supplementary material for: Isochrony in barks of Cape fur seal (Arctocephalus pusillus pusillus) pups and adults
Source: Ecol Evol. 2024 Mar 7;14(3):e11085. doi: 10.1002/ece3.11085 (PMC10920323; doi:10.1002/ece3.11085)
Supplement: Supplementary file 2 — Table S1 [file ECE3-14-e11085-s002.docx]

| **ID** | **Age class** | **Unbiased CV** | **nPVI** | **IOI beat in Hz** | **Beat precision** | **CV of beat precision** | **nPVI of beat precision** |
| --- | --- | --- | --- | --- | --- | --- | --- |
| 1 | adult | 0.166042 | 21.88141 | 3.701465 | 0.736042 | 0.396446 | 46.1271 |
| 2 | adult | 0.071303 | 6.869989 | 2.933672 | 0.548777 | 0.495962 | 37.71097 |
| 3 | adult | 0.06971 | 6.582492 | 3.099468 | 0.488452 | 0.618432 | 48.55741 |
| 4 | adult | 0.062475 | 8.6888 | 2.976726 | 0.782291 | 0.278933 | 18.75284 |
| 5 | adult | 0.064128 | 9.739604 | 3.264167 | 0.827563 | 0.272135 | 21.5268 |
| 6 | adult | 0.053272 | 7.559108 | 2.742798 | 0.324272 | 0.824969 | 47.28494 |
| 7 | adult | 0.080582 | 26.27517 | 3.798869 | 0.761787 | 0.312167 | 37.49993 |
| 8 | adult | 0.068669 | 9.039279 | 3.582766 | 0.792187 | 0.228765 | 23.92028 |
| 9 | adult | 0.143394 | N/A | 3.423873 | 0.748583 | 0.411786 | 69.64847 |
| 10 | adult | 0.083693 | 13.73179 | 3.54102 | 0.524107 | 0.471916 | 28.53383 |
| 11 | adult | 0.061685 | 7.418506 | 2.407121 | 0.341206 | 0.985883 | 52.5258 |
| 12 | adult | 0.04173 | 4.349899 | 2.866216 | 0.538199 | 0.557935 | 40.5227 |
| 13 | adult | 0.072546 | 8.645064 | 3.848043 | 0.816365 | 0.267785 | 20.39435 |
| 14 | adult | 0.046999 | 5.30905 | 3.167264 | 0.578238 | 0.483419 | 37.81078 |
| 15 | adult | 0.04184 | 5.851668 | 2.31674 | 0.460917 | 0.734109 | 44.08972 |
| 16 | adult | 0.091784 | 8.428586 | 3.70664 | 0.850722 | 0.218388 | 24.46998 |
| 17 | adult | 0.058647 | 4.586057 | 3.401907 | 0.259307 | 0.866125 | 49.20419 |
| 18 | pup | 0.66233 | 28.72199 | 2.38851 | 0.402957 | 0.912928 | 135.6951 |
| 19 | pup | 0.68198 | 193.1804 | 1.538102 | 0.151366 | 1.166282 | 247.1537 |
| 20 | pup | 0.054879 | 10.0543 | 2.756205 | 0.585847 | 0.463442 | 33.41281 |
| 21 | pup | 0.499139 | 45.79454 | 3.570274 | 0.471705 | 0.81851 | 197.4651 |
| 22 | pup | 0.079725 | 10.38943 | 3.026895 | 0.40452 | 0.636403 | 35.51763 |
| 23 | pup | 0.116625 | 24.07852 | 3.878012 | 0.763736 | 0.255322 | 30.82784 |
| 24 | pup | 0.055108 | 3.833614 | 3.216784 | 0.881849 | 0.258297 | 13.95499 |
| 25 | pup | 0.050086 | 6.353673 | 3.655097 | 0.797573 | 0.222958 | 20.81216 |
| 26 | pup | 0.056501 | 8.939387 | 3.140797 | 0.635324 | 0.420208 | 29.26158 |
| 27 | pup | 0.456862 | 73.82912 | 2.58763 | 0.438004 | 1.00884 | 166.9318 |
| 28 | pup | 0.335463 | 33.61327 | 3.139721 | 0.462132 | 0.743659 | 72.86555 |
| 29 | pup | 0.241362 | 19.67618 | 3.27967 | 0.402603 | 0.748101 | 103.8226 |
| 30 | pup | 0.654243 | 98.10309 | 1.093824 | 0.336441 | 1.084348 | 138.857 |
| 31 | pup | 0.09575 | 5.98213 | 3.005533 | 0.153093 | 1.413468 | 112.7945 |
| 32 | pup | 0.104896 | 12.19869 | 2.892837 | 0.445959 | 0.729093 | 64.33729 |
| 33 | pup | 0.065439 | 6.584467 | 2.546374 | 0.420842 | 0.795153 | 44.83731 |
| 34 | pup | 0.037 | N/A | 3.27967 | 0.655196 | 0.504873 | 81.72567 |

**Supplementary Table 1.** **Rhythm indices calculated for bark sequences of adults and pups. Indices correspond to points 2-7 described in the methods section.**
