# Supplementary material for: Isochrony in barks of Cape fur seal (Arctocephalus pusillus pusillus) pups and adults
Source: Ecol Evol. 2024 Mar 7;14(3):e11085. doi: 10.1002/ece3.11085 (PMC10920323; doi:10.1002/ece3.11085)
Supplement: Supplementary file 3 — Table S2 [file ECE3-14-e11085-s004.docx]

| **Index** | **ID** | **Age class** | **Integer ratio (rk)** | **Index** | **ID** | **Age class** | **Integer ratio (rk)** | **Index** | **ID** | **Age class** | **Integer ratio (rk)** |
| --- | --- | --- | --- | --- | --- | --- | --- | --- | --- | --- | --- |
| 1 | 1 | adult | 0.55 | 60 | 13 | adult | 0.52641 | 119 | 24 | pup | 0.492188 |
| 2 | 1 | adult | 0.529412 | 61 | 13 | adult | 0.460615 | 120 | 24 | pup | 0.507812 |
| 3 | 1 | adult | 0.48855 | 62 | 14 | adult | 0.487039 | 121 | 24 | pup | 0.5 |
| 4 | 1 | adult | 0.426752 | 63 | 14 | adult | 0.494736 | 122 | 24 | pup | 0.504 |
| 5 | 2 | adult | 0.471503 | 64 | 14 | adult | 0.49168 | 123 | 24 | pup | 0.504065 |
| 6 | 2 | adult | 0.497561 | 65 | 15 | adult | 0.478852 | 124 | 24 | pup | 0.516949 |
| 7 | 2 | adult | 0.497585 | 66 | 15 | adult | 0.511111 | 125 | 24 | pup | 0.5 |
| 8 | 2 | adult | 0.53886 | 67 | 15 | adult | 0.510836 | 126 | 24 | pup | 0.483051 |
| 9 | 2 | adult | 0.497207 | 68 | 15 | adult | 0.500792 | 127 | 25 | pup | 0.498392 |
| 10 | 2 | adult | 0.48913 | 69 | 16 | adult | 0.505134 | 128 | 25 | pup | 0.501608 |
| 11 | 3 | adult | 0.502959 | 70 | 16 | adult | 0.495561 | 129 | 25 | pup | 0.525424 |
| 12 | 3 | adult | 0.464088 | 71 | 16 | adult | 0.499305 | 130 | 25 | pup | 0.468227 |
| 13 | 3 | adult | 0.507853 | 72 | 16 | adult | 0.49697 | 131 | 25 | pup | 0.496875 |
| 14 | 3 | adult | 0.497354 | 73 | 16 | adult | 0.55505 | 132 | 26 | pup | 0.477273 |
| 15 | 4 | adult | 0.538462 | 74 | 17 | adult | 0.483193 | 133 | 26 | pup | 0.528736 |
| 16 | 4 | adult | 0.477273 | 75 | 17 | adult | 0.501018 | 134 | 26 | pup | 0.482353 |
| 17 | 4 | adult | 0.496403 | 76 | 17 | adult | 0.505156 | 135 | 26 | pup | 0.503817 |
| 18 | 4 | adult | 0.520446 | 77 | 17 | adult | 0.475246 | 136 | 26 | pup | 0.526316 |
| 19 | 4 | adult | 0.507874 | 78 | 17 | adult | 0.509615 | 137 | 26 | pup | 0.4875 |
| 20 | 4 | adult | 0.484496 | 79 | 17 | adult | 0.505952 | 138 | 27 | pup | 0.515152 |
| 21 | 5 | adult | 0.515748 | 80 | 17 | adult | 0.501006 | 139 | 27 | pup | 0.330579 |
| 22 | 5 | adult | 0.506173 | 81 | 17 | adult | 0.474189 | 140 | 28 | pup | 0.493151 |
| 23 | 5 | adult | 0.519481 | 82 | 17 | adult | 0.509258 | 141 | 28 | pup | 0.486842 |
| 24 | 5 | adult | 0.468354 | 83 | 17 | adult | 0.490741 | 142 | 28 | pup | 0.345133 |
| 25 | 6 | adult | 0.488136 | 84 | 17 | adult | 0.514019 | 143 | 28 | pup | 0.646288 |
| 26 | 6 | adult | 0.513605 | 85 | 17 | adult | 0.487084 | 144 | 28 | pup | 0.48503 |
| 27 | 6 | adult | 0.510714 | 86 | 17 | adult | 0.49799 | 145 | 29 | pup | 0.490323 |
| 28 | 6 | adult | 0.485816 | 87 | 18 | pup | 0.504348 | 146 | 29 | pup | 0.51634 |
| 29 | 6 | adult | 0.510563 | 88 | 18 | pup | 0.483051 | 147 | 29 | pup | 0.517483 |
| 30 | 6 | adult | 0.466443 | 89 | 18 | pup | 0.486056 | 148 | 29 | pup | 0.492857 |
| 31 | 7 | adult | 0.531707 | 90 | 18 | pup | 0.483146 | 149 | 29 | pup | 0.355 |
| 32 | 7 | adult | 0.466019 | 91 | 18 | pup | 0.264875 | 150 | 29 | pup | 0.58371 |
| 33 | 8 | adult | 0.486726 | 92 | 19 | pup | 0.684058 | 151 | 29 | pup | 0.484211 |
| 34 | 8 | adult | 0.513274 | 93 | 19 | pup | 0.201107 | 152 | 30 | pup | 0.579879 |
| 35 | 8 | adult | 0.486726 | 94 | 20 | pup | 0.512563 | 153 | 30 | pup | 0.674847 |
| 36 | 8 | adult | 0.495726 | 95 | 20 | pup | 0.473171 | 154 | 30 | pup | 0.62234 |
| 37 | 8 | adult | 0.546296 | 96 | 20 | pup | 0.529412 | 155 | 30 | pup | 0.210059 |
| 38 | 9 | adult | 0.545064 | 97 | 20 | pup | 0.484848 | 156 | 30 | pup | 0.814024 |
| 39 | 10 | adult | 0.556291 | 98 | 20 | pup | 0.483412 | 157 | 31 | pup | 0.493939 |
| 40 | 10 | adult | 0.478571 | 99 | 21 | pup | 0.468085 | 158 | 31 | pup | 0.485465 |
| 41 | 10 | adult | 0.480263 | 100 | 21 | pup | 0.316456 | 159 | 31 | pup | 0.491667 |
| 42 | 10 | adult | 0.523179 | 101 | 21 | pup | 0.486486 | 160 | 31 | pup | 0.482565 |
| 43 | 10 | adult | 0.464516 | 102 | 22 | pup | 0.447917 | 161 | 31 | pup | 0.495235 |
| 44 | 10 | adult | 0.515528 | 103 | 22 | pup | 0.524752 | 162 | 31 | pup | 0.515464 |
| 45 | 11 | adult | 0.489796 | 104 | 22 | pup | 0.545455 | 163 | 31 | pup | 0.489583 |
| 46 | 11 | adult | 0.511364 | 105 | 22 | pup | 0.47619 | 164 | 31 | pup | 0.472323 |
| 47 | 11 | adult | 0.505882 | 106 | 22 | pup | 0.483516 | 165 | 32 | pup | 0.529255 |
| 48 | 11 | adult | 0.486111 | 107 | 22 | pup | 0.497354 | 166 | 32 | pup | 0.502841 |
| 49 | 11 | adult | 0.468354 | 108 | 22 | pup | 0.487179 | 167 | 32 | pup | 0.464191 |
| 50 | 11 | adult | 0.527197 | 109 | 22 | pup | 0.529101 | 168 | 32 | pup | 0.476415 |
| 51 | 11 | adult | 0.488904 | 110 | 22 | pup | 0.491713 | 169 | 33 | pup | 0.46732 |
| 52 | 12 | adult | 0.497393 | 111 | 22 | pup | 0.481675 | 170 | 33 | pup | 0.493939 |
| 53 | 12 | adult | 0.494016 | 112 | 23 | pup | 0.56 | 171 | 33 | pup | 0.507599 |
| 54 | 12 | adult | 0.486842 | 113 | 23 | pup | 0.453608 | 172 | 33 | pup | 0.495413 |
| 55 | 13 | adult | 0.486811 | 114 | 23 | pup | 0.53 | 173 | 33 | pup | 0.522152 |
| 56 | 13 | adult | 0.485073 | 115 | 23 | pup | 0.449761 | 174 | 33 | pup | 0.509228 |
| 57 | 13 | adult | 0.518797 | 116 | 23 | pup | 0.558252 | 175 | 34 | pup | 0.488372 |
| 58 | 13 | adult | 0.507132 | 117 | 23 | pup | 0.443902 |  |  |  |  |
| 59 | 13 | adult | 0.509835 | 118 | 24 | pup | 0.519084 |  |  |  |  |

**Supplementary Table 2.** **Integer ratios (rk) calculated for each pair of consecutive calls in barking bouts of adults and pups. While these values do not differ significantly between the age classes, pups show significant deviations from the temporal pattern.**
