# Supplementary material for: Isochrony in barks of Cape fur seal (Arctocephalus pusillus pusillus) pups and adults
Source: Ecol Evol. 2024 Mar 7;14(3):e11085. doi: 10.1002/ece3.11085 (PMC10920323; doi:10.1002/ece3.11085)
Supplement: Supplementary file 4 — Appendix S1 [file ECE3-14-e11085-s001.docx]

Appendix S1

Supplementary Materials

The following supporting information can be downloaded at: <https://osf.io/6rn82/?view_only=5687781b6424423b99b01b7c98a8af16>, Audio S1: Bark sequences of six Cape fur seal pups (merged). Audio S2: Bark sequences of two adult Cape fur seal females (merged). Video S1: Mock fight between two Cape fur seal pups at Lamberts Bay, South Africa. Audio was removed because of poor quality, to avoid confusion with general colony noises. Courtesy of Tess Gridely. Table S1: Rhythm indices calculated for bark sequences of adults and pups. Table S2: Integer ratios (rk) calculated for each pair of consecutive calls in barking bouts of adults and pups.
